# Supplementary material for: Distinct forms of structural plasticity of adult-born interneuron spines in the mouse olfactory bulb induced by different odor learning paradigms
Source: Commun Biol. 2024 Apr 6;7:420. doi: 10.1038/s42003-024-06115-7 (PMC10998910; doi:10.1038/s42003-024-06115-7)
Supplement: Supplementary file 1 — Supplementary Information [file 42003_2024_6115_MOESM1_ESM.pdf]

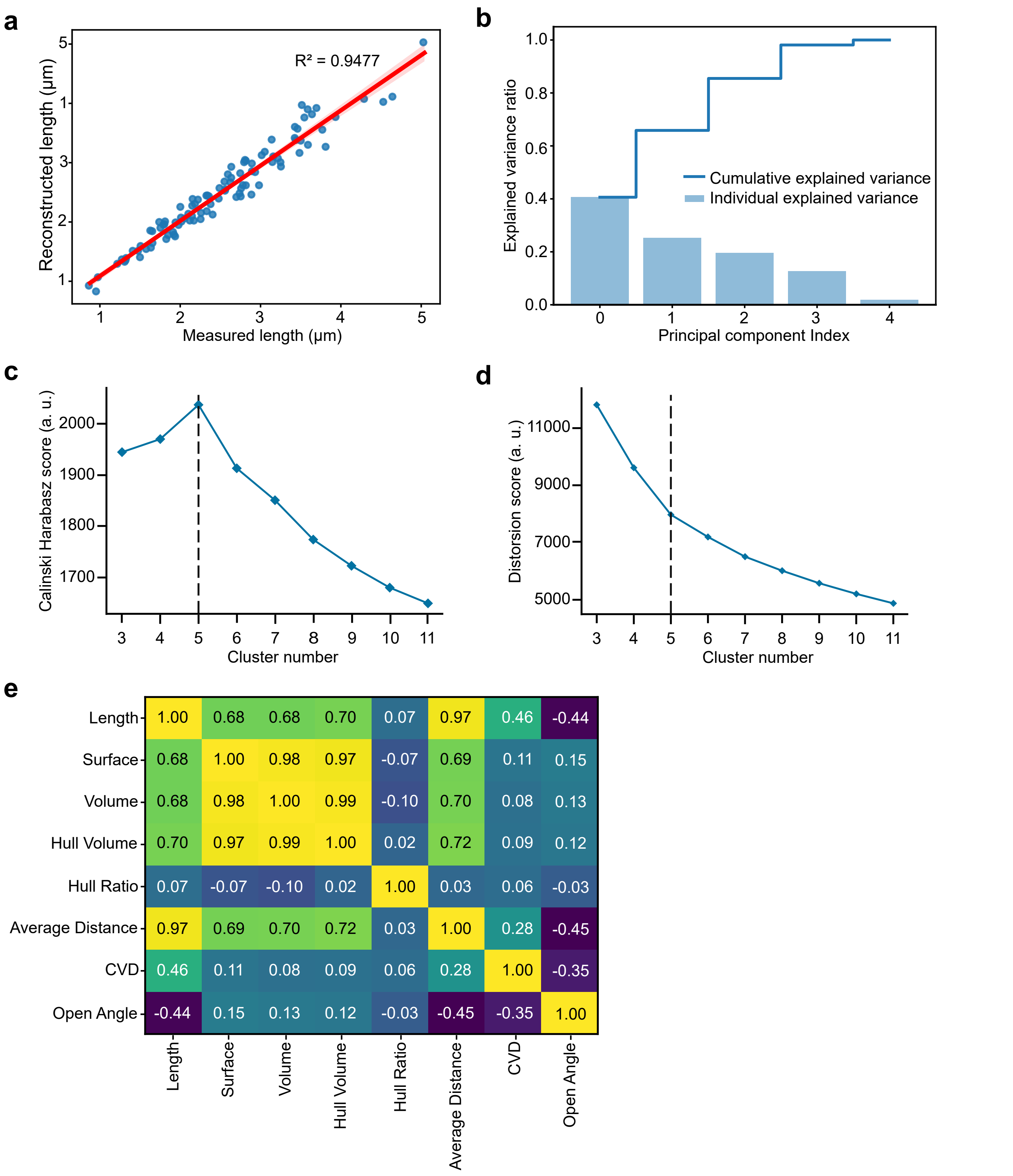

Supplementary Figure 1. Comprehensive analysis of dendritic spines dataset: correlation, variance, clustering, and morphometric feature interrelations. (**a**). A correlation factor (R^2) of 0.9477 was found between dendritic spine measurements obtained through expert blind assessment and automated quantification through the established pipeline. (**b**) The cumulative explained variance represents the total variance accumulated across successive principal components. In contrast, the individual explained variance relates to the amount of variance attributed to each separate principal component. (**c**) The Calinski-Harabasz score and (**d**) the Elbow score showed that the most likely number of clusters was 5. (**e**) Pearson's correlation coefficients between the morphometric features: Length, Surface, Volume, Hull Volume, Hull Ratio, Average Distance, CVD, and Open Angle.

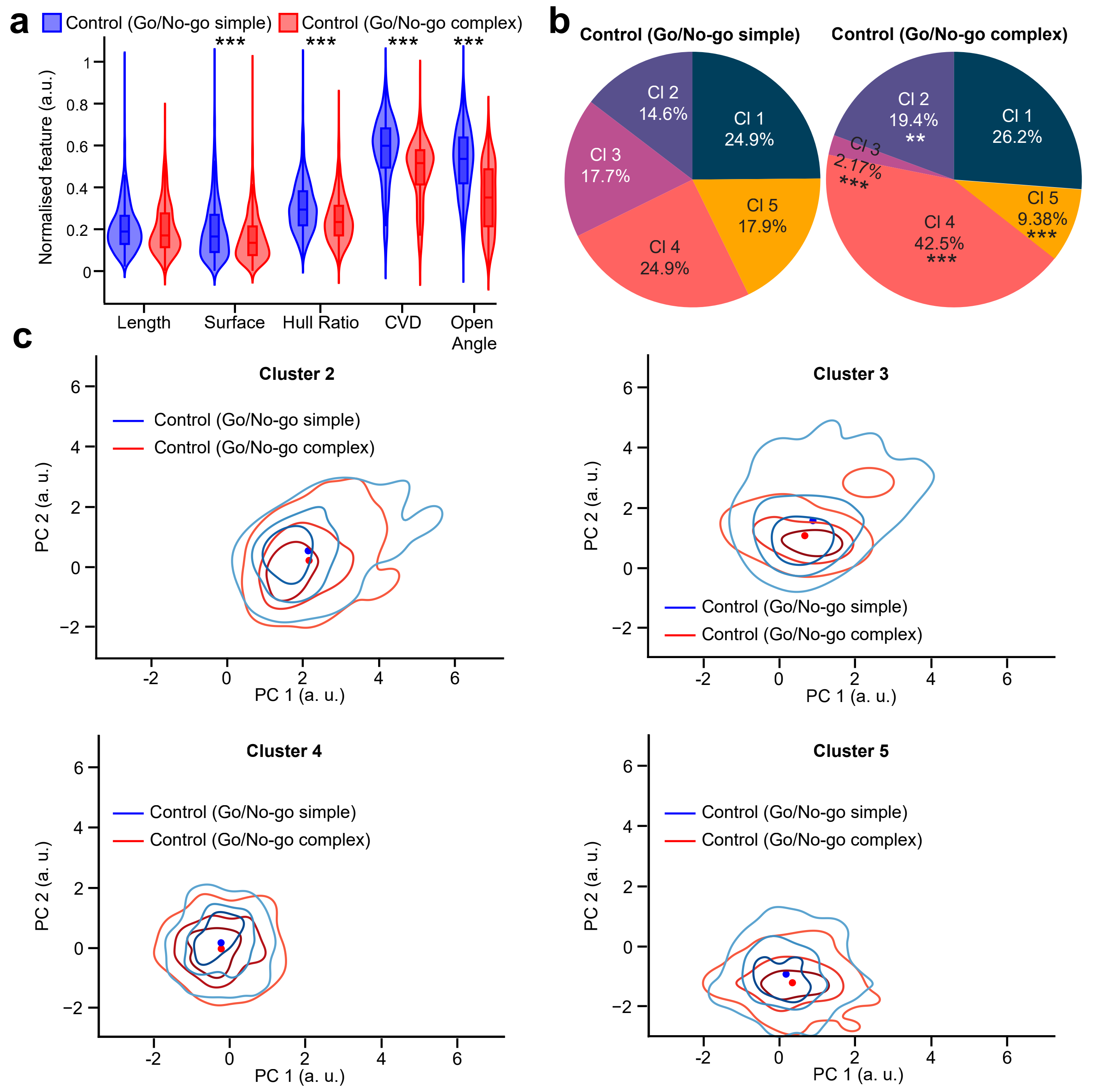

Supplementary Figure 2. Morphological differences of spines between the control groups of the simple and complex go/no-go tasks. (**a**) Violin plot representation of the control group of the simple task in blue and the control group of the complex task in red for Length, Surface, Hull Ratio, CVD, and Open Angle. With an unpaired Student t-test, p-values: 0.15 for Length and <0.001 for Surface, Hull ratio, CVD and Open angle. (**b**) Pie chart representation of the cluster distribution in the control group of the simple task and the control group of the complex task. With Agresti-Caffo independence test: 0.48, 0.0044, <0.001, <0.001, < 0.001 for clusters 1, 2, 3, 4, 5, respectively. (**c**) KDE map of the representation of the spine densities of clusters 2, 3, 4, and 5 in the space of PC1 and PC2 for the control group of the simple task in blue and the control of the complex task in red.

*** p<0.001 with a Student t-test.
