# Supplementary material for: Distinct forms of structural plasticity of adult-born interneuron spines in the mouse olfactory bulb induced by different odor learning paradigms
Source: Commun Biol. 2024 Apr 6;7:420. doi: 10.1038/s42003-024-06115-7 (PMC10998910; doi:10.1038/s42003-024-06115-7)
Supplement: Supplementary file 2 — Description of Additional Supplementary Files [file 42003_2024_6115_MOESM2_ESM.pdf]

Description of Additional Supplementary Files

File name: Supplementary Data 1
Description: The source data for the graphs in the paper.
